# Impairing protein–protein interactions in an essential tRNA modification complex: An innovative antimicrobial strategy against *Pseudomonas aeruginosa*

**DOI:** 10.1002/psc.3658

**Published:** 2024-10-22

**Authors:** Michela Bollati, Elettra Fasola, Stefano Pieraccini, Francesca Freddi, Paolo Cocomazzi, Francesco Oliva, Merlin Klußmann, Angelo Maspero, Umberto Piarulli, Silvia Ferrara, Sara Pellegrino, Giovanni Bertoni, Silvia Gazzola

**Affiliations:** ^1^ Institute of Biophysics National Research Council Milan Italy; ^2^ Department of Biosciences Università degli Studi di Milano Milan Italy; ^3^ Department of Science and High Technology Università degli Studi dell'Insubria Como Italy; ^4^ Department of Chemistry Università degli Studi di Milano Milan Italy; ^5^ Department of Chemistry, Institute for Biochemistry University of Cologne Cologne Germany; ^6^ Pharmaceutical Science Department University of Milan Milan Italy

**Keywords:** cell‐penetrating peptide, PPIs, protein‐mimetic peptide, protein–protein interactions, *Pseudomonas aeruginosa*, TC‐complex, YeaZ

## Abstract

Protein–protein interactions (PPIs) have been recognized as a promising target for the development of new drugs, as proved by the growing number of PPI modulators reaching clinical trials. In this context, peptides represent a valid alternative to small molecules, owing to their unique ability to mimic the target protein structure and interact with wider surface areas. Among the possible fields of interest, bacterial PPIs represent an attractive target to face the urgent necessity to fight antibiotic resistance. Growing attention has been paid to the YgjD/YeaZ/YjeE complex responsible for the essential t^6^A_37_ tRNA modification in bacteria. We previously identified an α‐helix on the surface of *Pseudomonas aeruginosa* YeaZ, crucial for the YeaZ‐YeaZ homodimer formation and the conserved YeaZ‐YgjD interactions. Herein, we present our studies for impairing the PPIs involved in the formation of the YeaZ dimers through synthetic peptide derivatives of this helical moiety, both *in vitro* with purified components and on *P. aeruginosa* cells. Our results proved the possibility of targeting those PPIs which are usually essential for protein functioning and thus are refractory to mutational changes and antibiotic resistance development.

## Graphical abstract

The design and synthesis of protein‐mimetic peptides (PMPs) for impairing protein–protein‐interactions of an essential tRNA modification complex in *Pseudomonas aeruginosa* (PAE) were investigated. **PMP3**, bearing a cell‐penetrating peptide, exhibits a significant concentration‐dependent effect on the growth rates of PAE, making it an interesting starting point for further optimization.

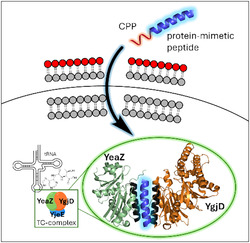

**Abbreviations**
CPP: cell‐penetrating peptide
PAE: *P. aeruginosa*
PMP: protein‐mimetic peptide
PPIs: protein–protein interactions
TC‐complex: threonylcarbamoyladenosine complex
WHO: World Health Organization

## 1 INTRODUCTION

Due to their fundamental role in regulating critical biological processes and transmembrane signal transduction in cells,^1^, ^2^ protein–protein interactions (PPIs) have been recently recognized as a promising class of biological targets for the development of new therapeutic agents for a wide number of diseases.^3^, ^4^ Indeed, in the past few decades, a growing number of PPI modulators have reached promising results in clinical trials and on the market, demonstrating the broad prospect of this new class of drugs.^5^, ^6^ However, the modulation of PPIs through small molecules is generally considered difficult^7^, ^8^ because of the usually large and mainly flat surfaces involved in the PPI, lacking suitable grooves or pockets for ligands to bind to. As an alternative, peptides offer the possibility to tackle these limitations as they closely mimic the features of a protein, but at the same time they are smaller, easier to synthesize, and punctually modifiable to improve the drug‐like properties.^9^, ^10^ Since peptides targeting PPIs are generally bigger than small molecules, they can interact with a larger surface, and this feature is particularly important when the drug‐resistance effect is easily developed in a specific disease.^11^, ^12^ For instance, particular attention is currently given to the development of novel antibacterial drugs with unconventional targets, like essential PPIs,^13^ due to the dramatic rise in the percentage of antibiotic‐resistant bacterial infections.^14^ Targeting bacterial PPIs is indeed a growing research area,^15^ which is supported by the fact that: (i) bacteria are less prone to develop antibiotic resistance thanks to the larger interaction surface between the target and the inhibitor, and (ii) bacterial PPIs are often absent or substantially different in the eukaryotic hosts.^16^

**FIGURE 1:**
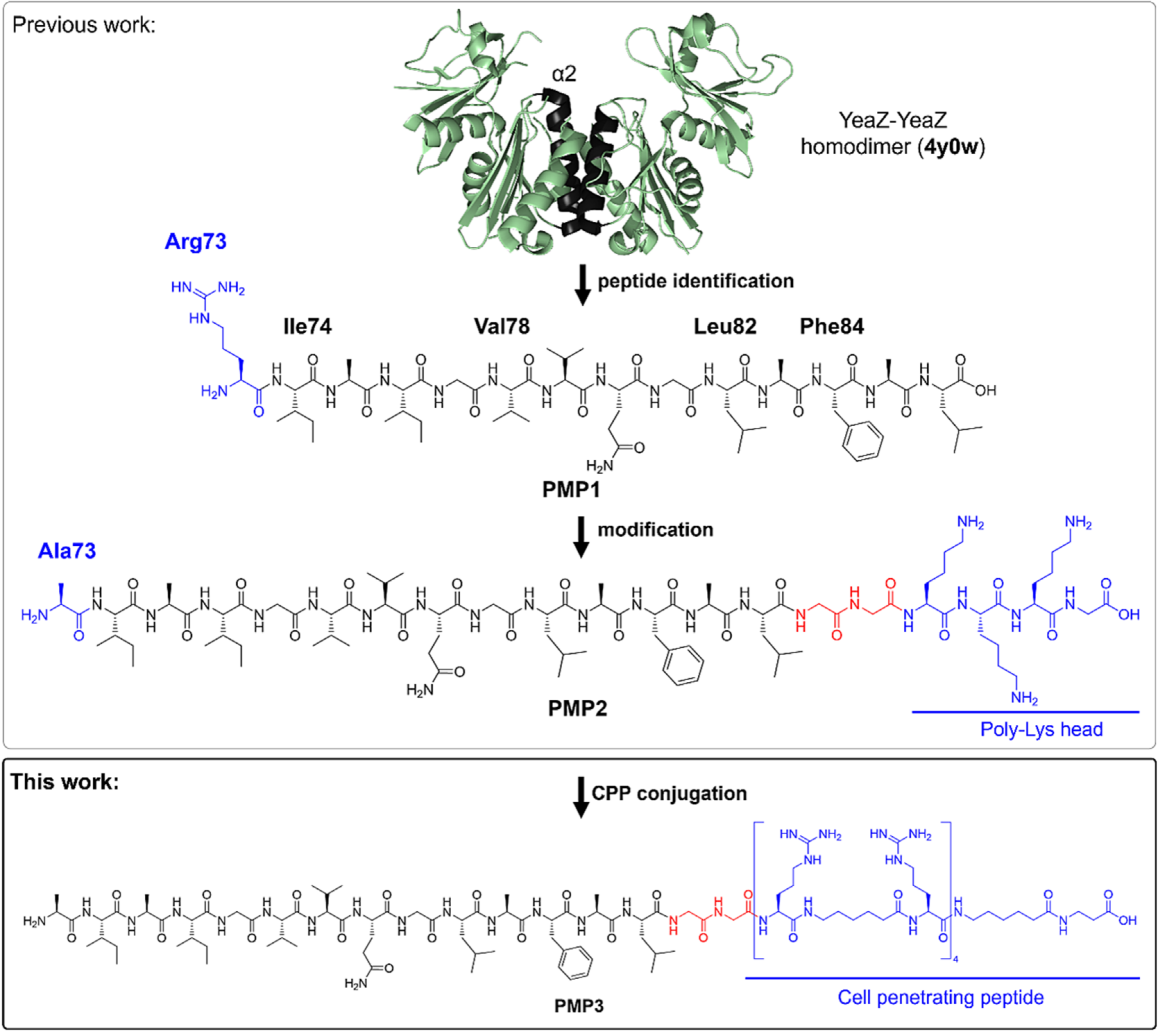
Previously identified protein‐mimetic‐peptide **PMP1** (in black) with its hot spots, and its further modifications connected through a Gly‐Gly spacer (in red), to obtain **PMP2** (previous work) and **PMP3** (this work).

*P. aeruginosa* (PAE) is a Gram‐negative bacterium that has been ranked by WHO as one out of three pathogens with the highest global priority for the development of novel effective antibacterials.^17^ PAE is a major cause of both acute and chronic lung infections, which develop in up to 60%–70% of cystic fibrosis (CF) patients, often leading to a more rapid decline of lung function that turns into greater morbidity and mortality.^18^, ^19^, ^20^ The misuse of classical antibiotics, whose mechanism of action is usually based on the interaction with a well‐defined ligand binding site, led to an ever‐increasing diffusion of PAE‐resistant strains. In this context, the production of new antibiotics is not only necessary but also urgent. The macromolecular threonylcarbamoyladenosine complex (TC‐complex) YgjD/YeaZ/YjeE has been proposed as a suitable target for such a purpose. This complex is devoted to the biosynthesis of N(6)‐threonylcarbamoyladenosine (t^6^A_37_), a modification in the anticodon stem‐loop of many tRNAs decoding ANN codons (N is any nucleotide), which is essential for the survival of bacteria.^21^, ^22^, ^23^ The YeaZ protein, specific to bacteria, plays a pivotal role in the recruitment of YgjD and YjeE and was recently proposed as a resuscitation‐promoting factor involved in the recovery from the VBNC state, i.e. the viable but nonculturable state. The high interest in these novel targets is documented by the increasing number of biochemical and structural studies performed to assess the mode of action and the protein–protein interactions (PPIs) involved in the YgjD/YeaZ/YjeE complex. YeaZ and YgjD form a strong binary dimer, which constitutes the minimal tRNA binding platform that carries out the transfer reaction. The interference with their interactions may thus strongly alter the viability of the bacterium. Moreover, since the residues involved in the PPIs are essential, antibacterials targeting these hot spots are expected to be long‐lived because potential resistance mutation(s) would likely lead to dead‐end protein complexes. Thanks to the solved crystal structure of the YeaZ‐YeaZ homodimer (PDB code 4Y0W),^21^ we recently reported the elucidation of the assembling mode of YeaZ protein through computational studies, and the identification of the protein‐mimetic peptide sequence AIAIGVVQGLAFAL (**PMP1**), primarily responsible for the dimer formation within a shorter fragment of the α2 helical domain (Arg73‐Leu86). Indeed, the YeaZ homodimer is mainly formed through the interaction of the two α2 α‐helices to finally generate a coiled‐coil‐like structure. By the addition of a poly‐Lys head, the highly hydrophobic structure **PMP1** was then modified to obtain an amphiphilic sequence (**PMP2**) capable of self‐assembling in helical nanofibers and 2D sheets as well as forming hydrogels in a controlled manner, because of a fine‐tuning of the environmental conditions (Figure 1).^24^

Since the PPIs and hot spots involved in the PAE YeaZ homodimer are conserved in the YeaZ/YgjD heterodimer,^21^, ^25^, ^26^ we hypothesized that **PMP1** might also represent a good starting point to target the YeaZ/YgjD interface. In this work, we report our studies focused on the PPIs involved in the formation of the TC‐complex and their possible inhibition by **PMP1** derivatives. The final aim was the interference of t^6^A_37_ biosynthesis and, consequently, the achievement of bacterial growth impairment. Our results showed that the derivative **PMP2**, which previously proved to maintain helicity outside the protein environment,^21^ also interacts with the formation of the YeaZ‐YeaZ homodimer, as highlighted by tryptophan (Trp) fluorescence and size exclusion chromatography (SEC) on the purified YeaZ protein. However, experiments on PAE cells showed that **PMP2** was not able to interfere with PAE growth. This could be due to inefficient penetration through the bacterial cell envelope. Therefore, we replaced the poly‐Lys head in **PMP2** with a cell‐penetrating peptide (CPP), which was shown to be an efficient carrier of peptide nucleic acid (PNA) cargos through the PAE cell envelope.^27^ This substitution generated the conjugate **PMP3** that was shown to affect bacterial growth. To the best of our knowledge, this is the first report in which the formation of the essential tRNA modification TC‐complex is impaired by rational‐designed peptide‐based PPI inhibitors.

## 2 MATERIALS AND METHODS

### 2.1 Modeling and molecular dynamic simulations

The YeaZ‐protein structure was obtained from the protein data bank (PDB ID code 4Y0W).^21^ In silico mapping of the residues giving a significant energetic contribution in protein–protein complex formation has been described elsewhere.^24^ Here, we evaluate the interaction between a YeaZ monomer and the **PMP1** peptide. To this aim, we extracted one monomer and the subsequence of a second monomer corresponding to the **PMP1** sequence from the crystal structure. The simulation of the system was carried out using water as an explicit water solvent and periodic boundary conditions. The description of the protein was performed with amber99SB‐ILDN force field^28^ whereas TIP3P model^29^ was used for water. The system was submitted to 50,000 steps of geometry optimization using the steepest descent method. It was then equilibrated, maintaining cα to crystallographic positions, for 200 ps with the number of particles, system volume, and temperature (300 K) constant (NVT conditions), followed by a second equilibration of additional 200 ps in the NPT (number of particles, system pressure, and temperature constant) ensemble. Subsequently, an unrestrained 100 ns long productive phase molecular dynamics simulation in the NPT ensemble was performed with constant temperature and pressure (300 K, 1 bar, respectively) through the velocity rescale algorithm^30^ and the Berendsen barostat.^31^ A 14 Å cutoff was applied for non‐bonded interactions, while the long‐range electrostatic interactions were calculated employing the Particles Mesh Ewald algorithm.^32^ During the molecular dynamic simulations, all bond lengths were constrained to their equilibrium values with the LINCS algorithm,^33^ allowing a time step of 2 fs. GROMACS 5.0.7 program suite^34^ was used to carry out the simulations and the subsequent analysis. Five hundred snapshots were extracted from the last 20 ns of the dynamics of the YeaZ‐**PMP1** complex (one snapshot every 40 ps). The molecular mechanics/Poisson–Boltzmann surface area (MM/PBSA) approach^35^ was used to perform computational alanine scanning (CAS) to estimate the contribution of each of the amino acids at the **PMP1**‐YeaZ protein interface to the binding energy. ΔG of binding was calculated with the MM/PBSA approach as implemented in the GMXPBSA 2.0 suite.^36^ This protocol implicitly assumes that the conformation is not significantly affected by point mutations in the protein. The validity of this assumption in CAS has been widely confirmed in the literature when applied to PPIs. A dielectric constant of 2 was chosen for the protein interior.

### 2.2 Peptide synthesis and purification

Commercially available reagents and resins were used as purchased from Sigma‐Aldrich®, TCI, Fluorochem©, BLD Pharmatech GmbH, and HPLC grade solvents were employed. Peptides were synthesized by manual solid phase synthesis by the Fmoc‐strategy starting from Fmoc‐AA preloaded Wang resin (Fmoc‐β‐Ala Wang resin 133 mg, commercial loading: 0.7 mmol/g and Fmoc‐Leu Wang resin 167 mg, commercial loading: 0.6 mmol/g). All HPLC purifications were performed with SHIMADZU LC‐20AP equipped with a diode array UV detector and Phenomenex Fusion‐RP 80 Å column. High‐resolution masses were obtained with Thermo Fisher Scientific Orbitrap Exploris™ 120 equipped with UHPLC and C18 column. The UV traces were acquired by analytical HPLC SHIMADZU LC‐20AP equipped with a diode array UV detector and C18 column.

Peptide **PMP2** was synthesized according to our previous work,^24^ and the CPP was synthesized as previously reported by Ghosal.^27^ More specifically, **PMP2** as well as the new sequence **PMP3** were manually synthesized on preloaded Wang resin (0.1 mmol‐scale). Couplings were performed using preactivated amino acids (3 eq) pre‐activation with DIPEA (8 eq) and COMU (3 eq). In the case of Fmoc‐Arg(Boc)_2_‐OH, DIC (5 eq) and Oxyma (5 eq) were used in a double coupling (5 hours each). Fmoc deprotection was performed with 20% *v/v* piperidine in DMF (3x5 minutes).

Alternatively, **PMP3** and the CPP [R‐Ahx‐R]_4_‐Ahx‐β‐Ala (Ahx: 6‐aminohexanoic acid) were synthesized by microwave‐assisted automated peptide synthesis on preloaded Wang resin (0.1 mmol‐scale) with a Liberty Blue synthesizer. Couplings were performed at 75°C using 170 W for 15 seconds and then at 90°C using 40 W for 110 seconds. Amino acids concentration was 0.2 M in DMF. DIC and Oxyma were used as coupling reagents (respectively 0.5 and 1 M in DMF). In the case of Arg, a double coupling procedure was performed. The Fmoc deprotection was carried out using 20% piperidine in DMF at 75°C using 155 W for 15 seconds and then at 90°C using 50 W for 50 seconds.

Finally, the peptides were cleaved from the resin with a cocktail of TFA/TIS/H_2_O 95:2.5:2.5, for 60 minutes plus a second time for 30 minutes. The peptides were then precipitated in ice‐cold diethyl ether, centrifuged at 5000 rpm for 10 minutes, washed 5 times with fresh ether, and dried. The crude peptides were purified by RP‐HPLC with SHIMADZU LC‐20AP equipped with a diode array UV detector and Phenomenex Fusion‐RP 80 Å column and characterized by HRMS with Thermo Fisher Scientific Orbitrap ExplorisTM 120 equipped with UHPLC and C18 column. The purities of the **PMP2** and **PMP3** were analyzed by analytical HPLC SHIMADZU LC‐20AP equipped with diode array UV detector and C18 column; the purity and the mass of the CPP were obtained with ADAMAS C‐18 column from Sepachrom and Fisons MD800 spectrometer with electrospray ion trap on a Finnigan LCQ advantage Thermo‐spectrometer, respectively (see gradients, traces, and mass spectra in the Supporting Information). Table 1 shows the synthesized peptide sequences for this work, the respective yields, purities, and MS data.

**TABLE 1:** List of the peptides synthesized for this work, with relative yields, purities, and MS data.

Peptide	Sequence	Yield	Purity	*m/z* calculated	*m/z* found
**PMP2**	AIAIGVVQGLAFAL‐GG‐KKKG	60%	≥95%	[M+2H]^2+^: 949.575	[M+2H]^2+^: 950.082
**PMP3**	AIAIGVVQGLAFAL‐GG‐[R‐Ahx‐R]_4_‐Ahx‐β‐Ala	7%	≥95%	[M+5H]^5+^: 669.222 [M+6H]^6+^: 557.852 [M+7H]^7+^: 478.301 [M+8H]^8+^: 418.639	[M+ 5H]^5+^: 669.430 [M+6H]^6+^: 558.193 [M+7H]^7+^: 478.595 [M+8H]^8+^: 418.771

### 2.3 Circular dichroism analysis

CD spectra were acquired on a JASCO J‐715 spectropolarimeter in a 1‐mm quartz cuvette, under N_2_, between 260 and 185 nm, at 20°C with 0.5 nm intervals, and 3 measurements were accumulated (settings: 100 mdeg sensitivity, continuous scan mode, 50 nm/min scan speed, 2 s response, bandwidth 1.0 nm). The resulting signal was converted from ellipticity (mdeg) to molar ellipticity [Q] in deg · cm^2^ · dmol^−1^. Peptides were solubilized in 100% MilliQ water or 1:1 water/TFE at 20 μM concentration. The helical propensity [R] was calculated using the R‐value, *i.e*. the ratio between molar ellipticity values at 222 and 207 nm, where R = 1 is defined as a reference for an ideal α‐helix.

### 2.4 *In vitro* binding assays

#### 2.4.1 Expression and purification of PAE YeaZ

Briefly, the YeaZ protein was produced in *E. coli* M15[pREP4] strain using a pQE30‐*Pa*YeaZ plasmid. Cells were grown in LB medium at 37°C till the OD600 reached 0.6, then the protein expression was induced by isopropyl‐β‐D‐thiogalactoside (IPTG) at a final concentration of 0.1 mM for 5 h at 30°C. After cell collection, the pellet was resuspended in a lysis buffer containing 50 mM phosphate buffer, 150 mM NaCl, and 10 mM imidazole, pH 7.4. Cells were subsequently disrupted by a French press disruptor (20 bar pressure), and one tablet of protease inhibitor (Roche), 10 mg/ml DNase I, and 5 mg/ml Ribonuclease A were added to the solution. After centrifugation at 18,000 rpm for 45 min, the supernatant was collected, filtered, and loaded onto a Ni‐NTA Superflow (GE Healthcare Life Science). YeaZ was further purified using a Superdex 75 gel filtration column (GE Healthcare Life Science) in buffer 50 mM Hepes pH 7.4, 150 mM NaCl at a concentration of 0.5 mg/ml, in which freshly purified protein exists as a monomer in solution, and stored at −80°C.

#### 2.4.2 Trp fluorescence

Spectrofluorometric assays were performed on a Varian Cary Eclipse fluorescence spectrophotometer (Agilent Technologies) at 20°C. YeaZ was used at a final concentration of 0.065 mg/ml in buffer Hepes 50 mM pH 7.4, NaCl 500 mM, 10% glycerol (with 2% *v/v* DMSO as control), while the peptides were used at a final concentration of 200 μM. We used an excitation wavelength of 280 nm and registered the fluorescence emission spectra between 300 and 400 nm. Measures were performed in triplicates.

#### 2.4.3 Size exclusion chromatography

Analytical SEC experiments were performed on a Superdex75 Increase 10/300 Gl column (GE Healthcare Life Science) equilibrated in buffer Hepes 50 mM pH 7.4, NaCl 500 mM, 10% glycerol. YeaZ was used at a final concentration of 0.5 mg/ml (with the addition of 2% DMSO as control) and the peptides were added at a final concentration of 200 μM.

#### 2.4.4 Microscale thermophoresis assay

Microscale thermophoresis (MST) experiments were performed using a Monolith X apparatus (NanoTemper Technologies GmbH, Munich, Germany) and analyzed with the MO. Affinity Analysis software (NanoTemper Technologies GmbH). Briefly, YeaZ was labeled with the red fluorescent dye RED‐NHS 2° generation according to manufacturer instructions. MST measurements were performed in triplicates at 24°C on in Hepes 50 mM pH 7.4, NaCl 500 mM, 10% glycerol, and 0.05% Tween20, using premium‐treated glass capillaries and at a fixed concentration of 50 nM YeaZ. Results were merged and analyzed.

### 2.5 Bacterial growth inhibition assay

Growth inhibition experiments were performed with *P. aeruginosa* PAO1 strain^37^ and *Escherichia coli* K‐12 MG1655.^38^ Cells were grown overnight in 10 ml of LB medium at 37°C with shaking. Overnight cultures were washed twice with phosphate‐buffered saline (PBS) and inoculated to an optical density at 595 nm (OD_595_) of 0.05 in 10 ml of Mueller Hinton Broth (Sigma‐Aldrich). Aliquots (200 μl) of inoculated medium were distributed in triplicate (technical replicates) in a 96‐Well Cellstar® suspension culture plate (Greiner Bio‐One) and incubated in a Sunrise microplate reader (Tecan Group) at 37°C with constant orbital shaking and real‐time OD_595_ measurements every 15 min. Three final peptide concentrations were tested: 3, 10, and 30 μM, respectively. Controls were made by adding sterilized water, in the same amount of the added peptide volumes. Three independent replicates were performed. Data were reported by plotting, at each time point, the mean OD_595_ values of the three replicates with error bars indicating Standard Error of Mean (SEM).

## 3 RESULTS AND DISCUSSION

### 3.1 Modeling and molecular dynamics simulations of PMP1 with YeaZ protein

**FIGURE 2:**
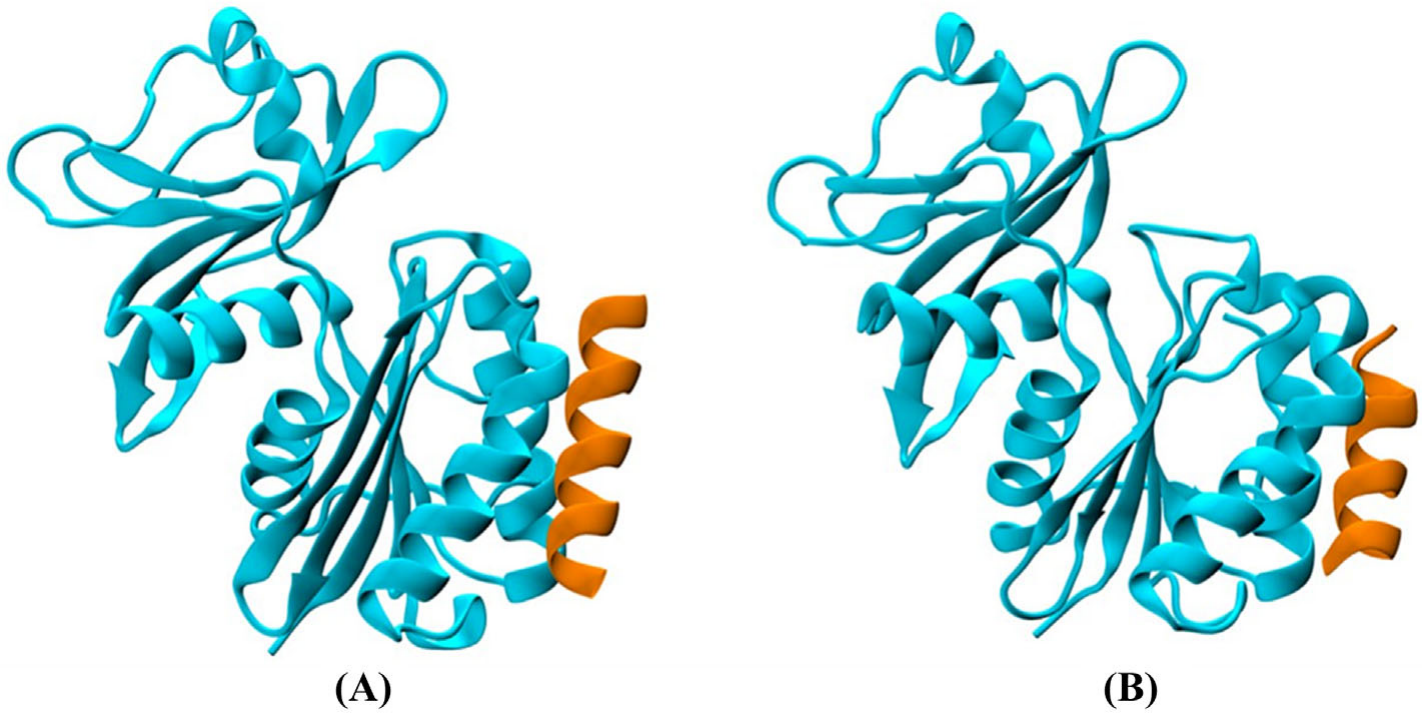
Structure of the YeaZ‐**PMP1** complex at the beginning (A) and at the end (B) of the 100 ns long MD simulation.

In principle, the binding capability of a protein‐mimetic peptide corresponding to a hot spots‐rich protein subsequence is not guaranteed because it may undergo major structural rearrangements when removed from the parent protein, and because of the complexity of the interaction networks leading to protein–protein complex formation. For this reason, we have run 100 ns long control molecular dynamics simulation of the YeaZ‐**PMP1** complex to investigate whether the identified peptide **PMP1** keeps its ability to bind to YeaZ, even when extracted from its protein environment. As shown in Figure 2, **PMP1** conserved its binding to YeaZ during the whole simulation, keeping also the helical structure exhibited within the parent protein.

In addition, a CAS analysis has been performed on **PMP1** to highlight whether hot spot residues are conserved relative to those detected in the YeaZ‐YeaZ complex. This technique allows us to calculate the difference in binding free energy (ΔΔ*G*) between the two protomers forming the analyzed complex (YeaZ‐YeaZ or YeaZ‐**PMP1**) upon mutation into the alanine of each of the interfacial residues. Among all the previously identified hot spots contributing to the YeaZ/YeaZ dimer formation,^24^ the residues Ile74, Val78, Leu82, and Phe84 belong also to the **PMP1** sequence, therefore, the CAS analysis was performed only on these hot spots to compare the ΔΔ*G* value obtained with the YeaZ‐YeaZ dimer and the YeaZ‐**PMP1** complex. As shown in Table 2, positive ΔΔ*G* values greater than 2 kcal/mol, like in the YeaZ dimer, were found when the respective residues Ile74, Val78, Leu82, and Phe84 on **PMP1** were replaced by alanine, suggesting that these punctual mutations reduce the binding energy in both types of complexes similarly. The relevant hot spots identified previously for the dimer formation are therefore conserved and are those contributing most to the YeaZ‐**PMP1** complex formation.

**TABLE 2:** Relevant hot spots (in bold) are conserved both in the YeaZ‐YeaZ and in the YeaZ‐**PMP1** complex.

YeaZ‐YeaZ		YeaZ‐PMP1	
Mutation	ΔΔ*G* (kcal·mol^−1^)	Mutation	ΔΔG (kcal·mol^−1^)
**Ile74Ala**	**3.5133**	**Ile74Ala**	**3.1309**
Ile76Ala	0.2629	Ile76Ala	1.1950
**Val78Ala**	**2.1032**	**Val78Ala**	**2.2227**
Gln80Ala	−0.1673	Gln80Ala	0.7887
**Leu82Ala**	**3.2982**	**Leu82Ala**	**2.8680**
**Phe84Ala**	**7.5524**	**Phe84Ala**	**6.8354**

These observations supported our hypothesis that **PMP1** should be able to bind to YeaZ and consequently reduce its ability to form protein complexes, thus impairing its function. Based on these results, and considering that the sequence **PMP1** is too hydrophobic as previously reported,^24^ we decided to test the derivative **PMP2** *in vitro* to evaluate its ability to inhibit the YeaZ‐YeaZ dimer formation, and subsequently on PAE cells to validate its ability to inhibit bacterial growth. Indeed, the **PMP2** peptide derives from the **PMP1** sequence RIAIGVVQGLAFAL (Arg73‐Leu86) and thus contains the four hot spots Ile74, Val78, Leu82, and Phe84 that mostly contribute to the interaction with the YeaZ protein. Additionally, the residue Arg73 was replaced with an Ala because of the negative ΔΔ*G* from CAS analysis (−13.07902 kcal/mol) performed in our previous work, and the cationic sequence KKK was added at C‐terminus to **PMP1** modified sequence through a GG‐spacer to give more stabilization to the α‐helix secondary structure and to enhance the solubility in water.^24^

### 3.2 *In vitro* binding assays of PMP2

**FIGURE 3:**
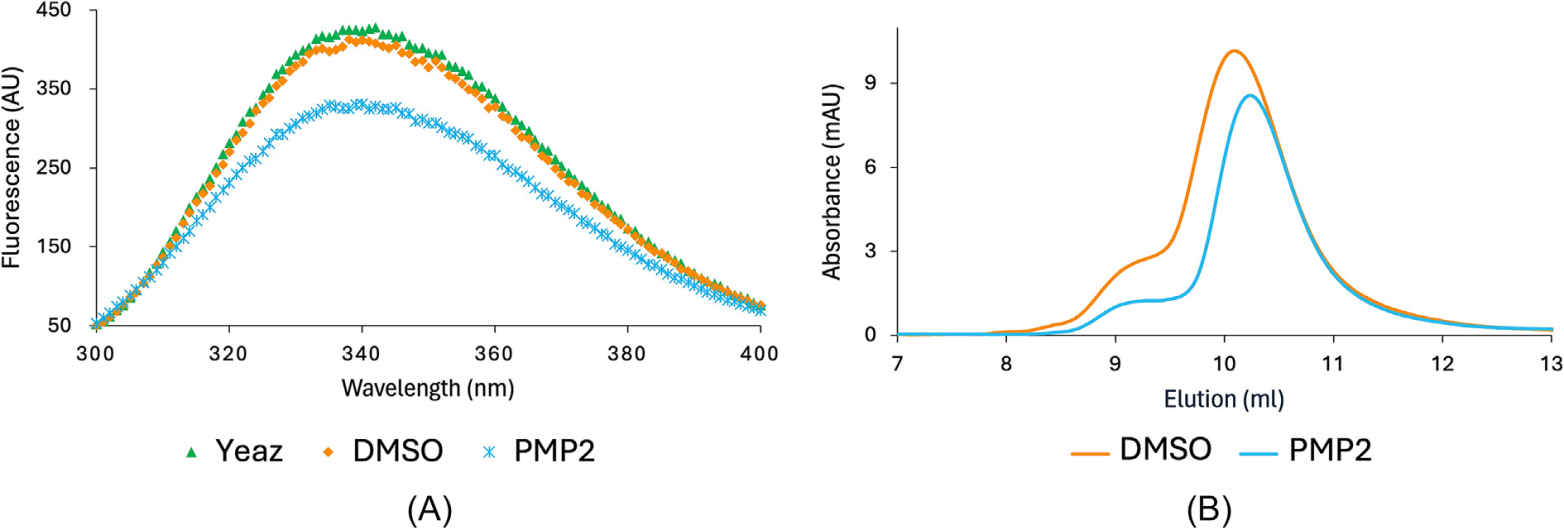
*In vitro* binding assays of **PMP2**. (A) Intrinsic tryptophan fluorescence emission spectra of YeaZ protein (green), in the presence of **PMP2** (blue), and 2% DMSO control (orange). (B) SEC elution profile of YeaZ with 2% DMSO control in orange and in the presence of **PMP2** in blue.

To evaluate the binding of **PMP2** in vitro, the recombinant His‐tagged YeaZ protein from PAE PAO1 was produced in *E. coli* and purified with final purity and yield values adequate for the binding studies. Compound **PMP2** was dissolved to 10mM with 100% DMSO and tested in in vitro assays at a final concentration of 200 μM (corresponding to a DMSO concentration of 2%). The interaction with the protein was first evaluated by exploiting the intrinsic fluorescence of the tryptophan residues (ITF) contained in the protein's amino acid sequence. The addition of 2% DMSO exerts only mild effects on the protein since the emission spectrum is comparable to the YeaZ sample without DMSO. The sample containing the peptide, however, showed a decreased fluorescence emission spectrum compared to the protein alone, suggesting an effect of the binding of **PMP2** to the protein (Figure 3A). Analytical size exclusion chromatography (SEC) was then run to evaluate the peptide's ability to disrupt or interfere with the formation of the homodimers. The YeaZ protein alone eluted at a volume corresponding mainly to its dimeric form (estimated molecular weight of 51 kDa of the main peak) and the effect of 2% v/v DMSO was irrelevant. The addition of **PMP2** caused a slight shift of the dimeric peak towards a smaller molecular weight (new estimated molecular weight of 49 kDa), suggesting an interaction between YeaZ and the peptide (Figure 3B). Unfortunately, above a concentration threshold, the results in ITF and SEC assays were biased by the poor solubility of **PMP2** in any tested solvent (H_2_O, 100% DMSO, and 10 mM NaOH). The formation of aggregates in solution at higher concentrations prevented the evaluation of a dose–response constant for the **PMP2** peptide.

Since the in vitro assays showed promising preliminary results in ITF and SEC, this prompted us to investigate the effects of the peptides directly on PAE cells.

### 3.3 Evaluation of PMP2 effects on PAE growth

**FIGURE 4:**
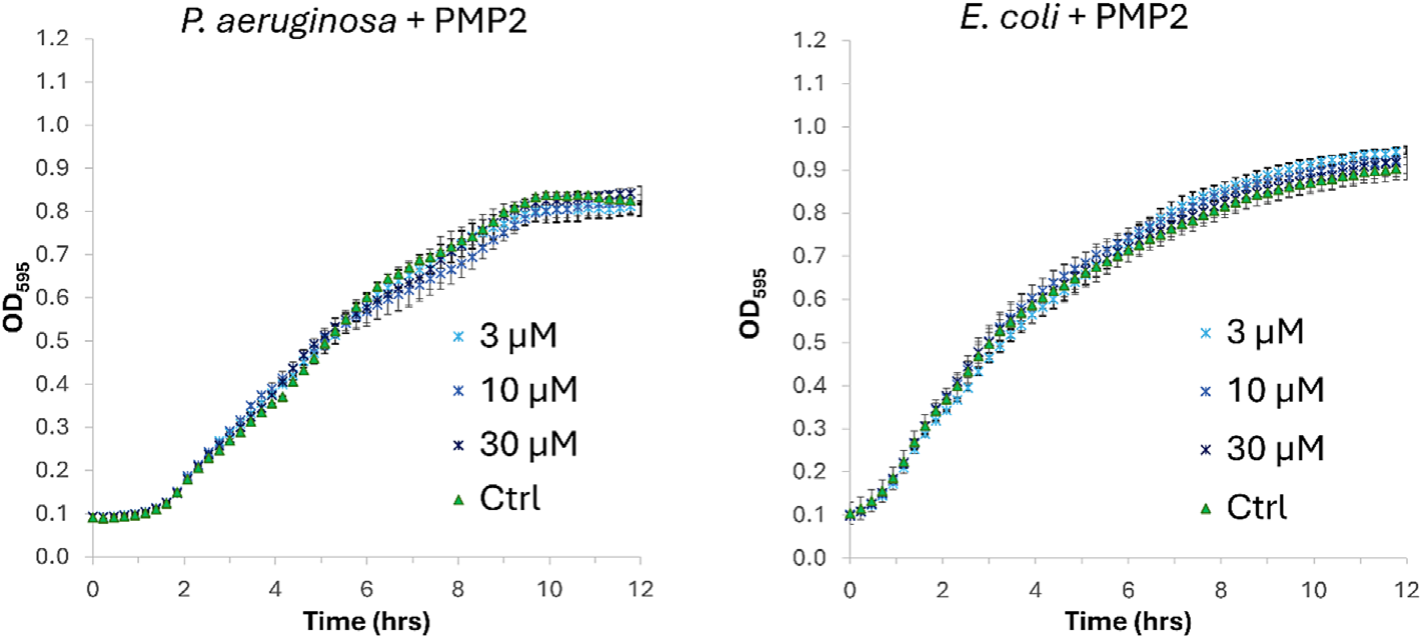
Growth curves of PAE and *E. coli* cells treated with increasing concentrations of **PMP2** (3, 10, and 30 μM). All data shown are mean values from three independent experiments. Error bars indicate the standard error of the mean (SEM).

To preliminarily study the effect of the peptide **PMP2** on bacterial cells, we monitored the growth of PAE PAO1 in the presence of increasing concentrations of the peptide (3 μM, 10 μM, 30 μM). Being the YeaZ protein conserved in bacteria, we also tested the compound on *E. coli* MG1655 cells. As shown in Figure 4, no effect of growth inhibition could be observed after treatment with **PMP2**, on both PAE PAO1 and *E. coli* MG1655 strains.

These negative results could be attributed to the lack of permeation of **PMP2** across the bacterial cell envelope, which could derive from its peculiar hydrophobicity and its tendency to aggregate at low concentrations. On the other hand, the intrinsic poor permeability of the Gram‐negative outer membrane is well known, and it indeed represents a real biological challenge, especially for peptide inhibitors.^39^

### 3.4 Conjugation of PMP1 with a cell‐penetrating peptide

**FIGURE 5:**
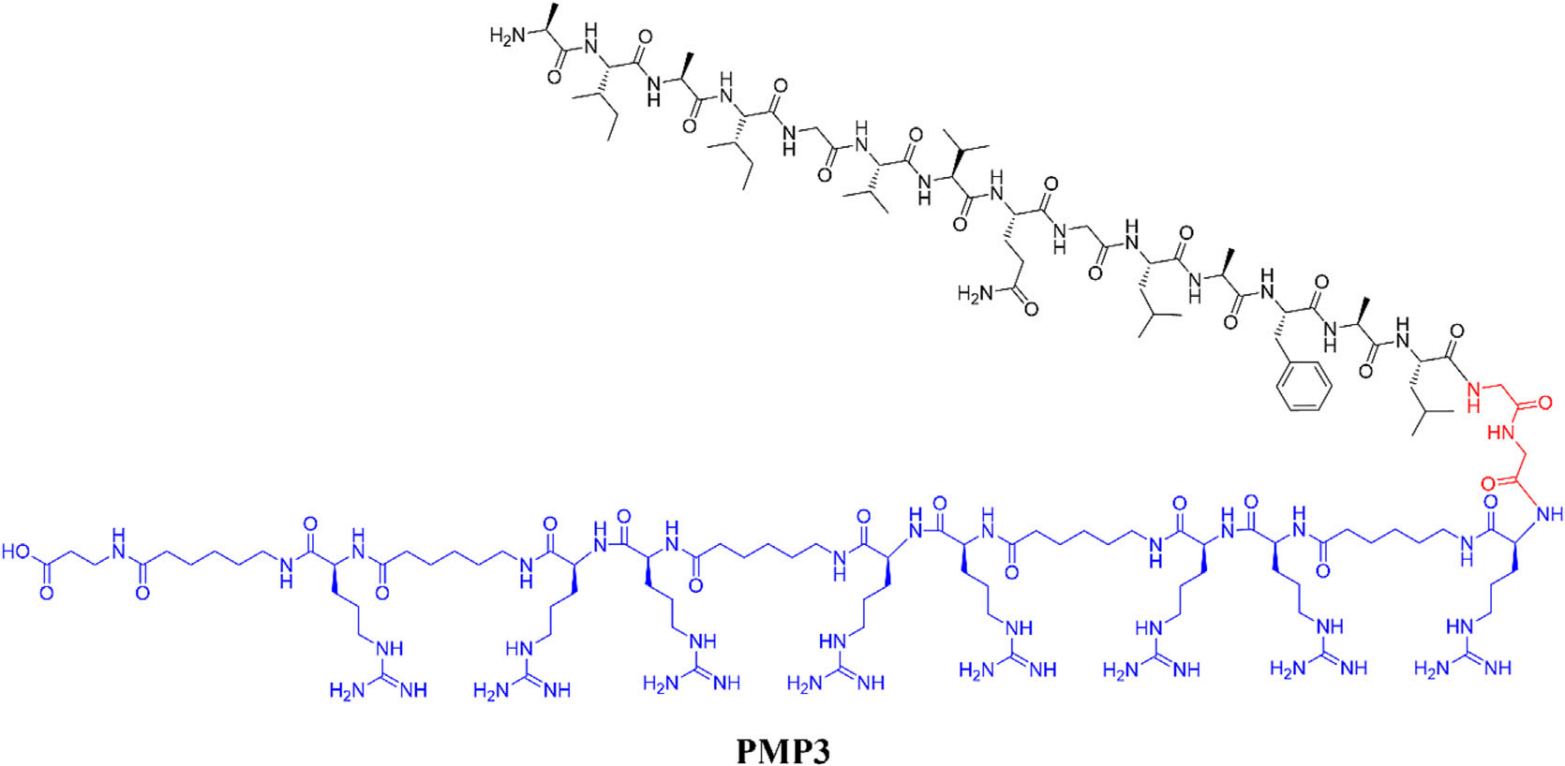
Chemical structure of the final **PMP3** conjugate, composed of the protein mimetic peptide **PMP1** bearing the modification Arg73Ala (in black) and the cell‐penetrating peptide (in blue), connected through a Gly‐Gly spacer (in red).

The design of peptide inhibitors that need to pass through the bacterial cell envelope is particularly arduous for Gram‐negative strains, and it often leads to obtain peptides showing little or no activity. Indeed, there are only a few reported antimicrobial peptides that are effective towards these bacteria.^39^ However, the interaction between **PMP2** and the YeaZ protein observed in *in vitro* experiments prompted us to investigate the possibility of conjugating the designed functional peptide with a suitable carrier able to cross the bacterial cell envelope. Among the various strategies reported in the literature such as liposomes and nanoparticles, cell‐penetrating peptides (CPPs) represent an attractive tool to deliver the inhibitor inside the bacterial cells.^40^ CPPs are usually positively charged sequences composed of Arg and/or Lys residues, able to interact with the negative phospholipid heads and glycosaminoglycans present on the membranes' surface.^41^ In 2012 Ghosal and co‐workers demonstrated that the synthetic CPP [R‐Ahx‐R]_4_‐Ahx‐β‐Ala^42^ was able to deliver antibacterial PNAs across the *P. aeruginosa* cell envelope. The final CPP‐PNA conjugates exhibited full inhibition of bacterial growth at 1–2 μM concentrations and without indications concerning bacterial membrane disruption.^27^ Later on, Amirkhanov and co‐workers demonstrated that the same CPP [R‐Ahx‐R]_4_‐Ahx‐β‐Ala^42^ does not show any antibacterial effect against PAE at a concentration below 40 μM,^43^ making it suitable to be used as a carrier to deliver our designed **PMP1** peptide into PAE cells to investigate its potential antibacterial effect. Thus, we synthesized the conjugate **PMP3**, composed by the modified sequence **PMP1** bearing the modification Arg73Ala connected at the C‐terminus through to the [R‐Ahx‐R]_4_‐Ahx‐β‐Ala CPP a Gly‐Gly spacer (Figure 5).

The choice of the conjugation at the C‐terminus rather than the N‐terminus of **PMP1** relies on the work of Ghosal and co‐workers,^27^ where they inserted the CPP [R‐Ahx‐R]_4_‐Ahx‐β‐Ala. Furthermore, we observed that the insertion of amino acid residues with the basic side chain at the C‐terminus of **PMP1** (i.e. the GGKKKG sequence present in **PMP2**) did not exert a negative influence on the α‐helical secondary structure formation.^24^

The peptide was synthesized by manual solid phase peptide synthesis using Fmoc‐strategy: COMU/DIPEA procedure was generally effective on couplings, except for Fmoc‐Arg(Boc)_2_‐OH, where DIC/Oxyma procedure was used. The synthesis afforded the compound with 7% of overall yield, due to multiple purification steps needed to reach a final purity above 95%.

**FIGURE 6:**
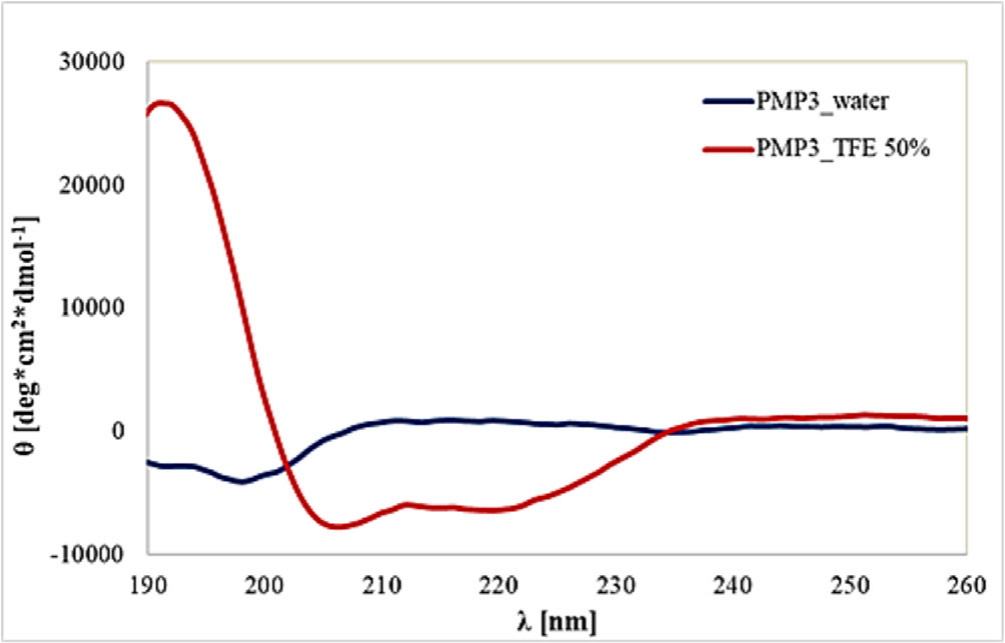
CD spectra of **PMP3** in 100% MilliQ water (blue), and in 1:1 water/TFE (red) at 20 uM concentration (R = 0.78).

The secondary structure of the newly synthesized **PMP3** was analyzed by circular dichroism by dissolving the peptide in 100% MilliQ water as well as in 1:1 water/TFE at 20 uM concentration. Although the peptide did not show any particular secondary structure in pure water, a clear α‐helix conformation was observed in the 1:1 water/TFE, demonstrating the high propensity of **PMP3** to fold into a helical structure in a more hydrophobic environment, that better mimics the protein background in which **PMP1** is naturally located (Figure 6).^44^

By contrast, the CD spectra of the CPP [R‐Ahx‐R]_4_‐Ahx‐β‐Ala did not show any specific conformation in the tested media, confirming that the helical contribution is mainly promoted by the **PMP1** sequence (see in the Supporting Information).

**FIGURE 7:**
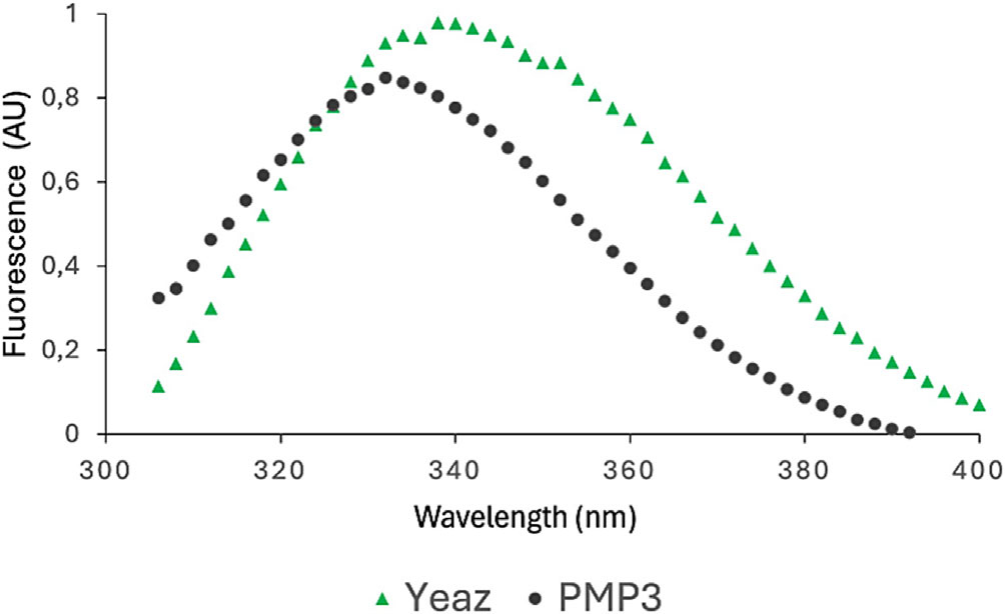
*In vitro* binding assays of **PMP3**. Intrinsic tryptophan fluorescence emission spectra of YeaZ protein (green) and in the presence of **PMP3** (black).

**PMP3** was then dissolved to 1mM in MilliQ water and evaluated *in vitro* in ITF assays. Interestingly, besides a reduction of the fluorescence intensity, a slight shift towards shorter wavelengths at around 330 nm could be observed, which suggested a change in the electric field around the tryptophans due to the interaction of the protein with the ligand (Figure 7). To further investigate the binding with **PMP3**, we performed a concentration‐response curve increasing the amounts of peptide. Unfortunately, above a concentration threshold, the intrinsic fluorescence of the peptide was higher than the intrinsic fluorescence of the YeaZ protein at 310–340 nm. We thus performed an MST assay exploiting the perturbation of thermal diffusion at 650 nm plotted against the peptide concentration. The MO. Affinity Analysis software detects a specific binding between **PMP3** and YeaZ, suggesting a *K*_d_ in the low micromolar. However, the precise value of the affinity constant could not be calculated since the concentration‐response curve did not reach the saturation plateau (see the Supporting Information).

**FIGURE 8:**
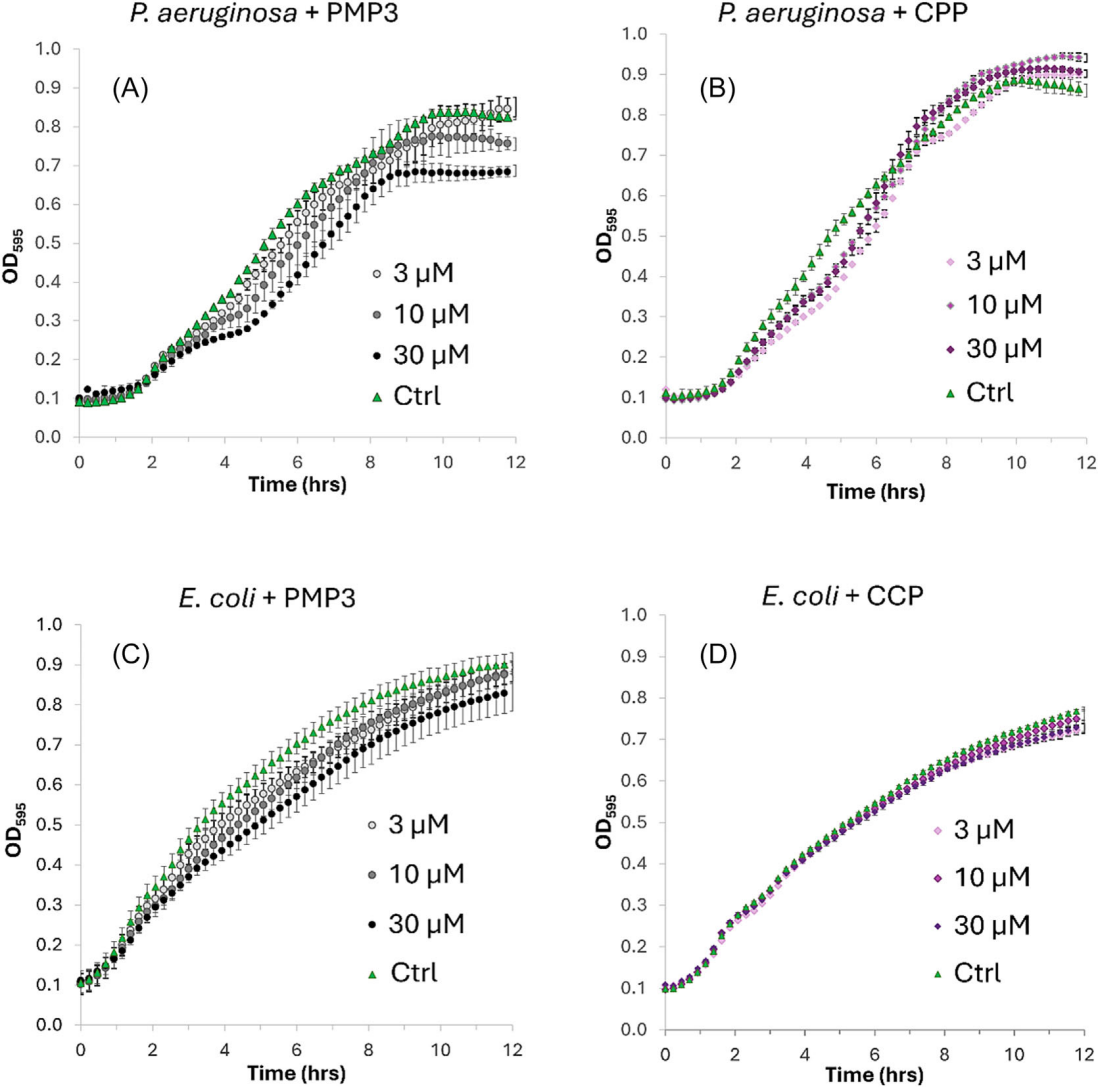
Growth curves of PAE (A) and *E. coli* (C) cells treated with increasing **PMP3** concentrations (3, 10, 30 μM) and with CCP alone (B) and (D), respectively. All data shown are mean values from three independent experiments. Error bars indicate the standard error of the mean (SEM).

Following these results, we evaluated the effects of **PMP3** on PAE PAO1 cells in the presence of increasing concentrations of the conjugate and the CPP alone, in the same conditions previously reported for **PMP2**. As shown in Figure 8A, PAO1 cells treated with **PMP3** showed a significant concentration‐dependent decrease in the growth rates. This inhibition pattern was not observed when the CPP alone was added at the same concentrations (Figure 8B), strongly suggesting that it does not contribute to growth inhibition, which is rather due to a genuine effect of the sequence of the functional peptide present in **PMP3**. Similarly, **PMP3** was incubated with *E. coli* MG1655 cells at 3, 10, and 30 μM, again using the CPP [R‐Ahx‐R]_4_‐Ahx‐β‐Ala alone as a control. As shown in Figure 8C, the **PMP3** effects on the growth inhibition of MG1655 were much weaker than those observed for PAO1, and not attributable to CCP (Figure 8D).

Although the YeaZ protein is present in *E. coli* as well,^45^ the primary structure is slightly different and lacks some of the hot spots identified in our in silico analysis for *P. aeruginosa* YeaZ. Ile74 and Leu82 are conserved, while Val78 and Phe84 are missing, which probably makes the peptide much more specific to YeaZ of PAE than that of *E. coli*.

## 4 CONCLUSIONS

Antibiotic resistance is to date one of the most urgent unsolved problems in the treatment of bacterial pathologies. Targeting bacterial PPIs has been proposed as an effective strategy to overcome the development of resistant bacterial strains since the larger surface involved in these interactions would require extensive mutations, which are less likely to be developed in bacteria. Based on the solved crystal structure of the PAE YeaZ‐YeaZ homodimer (PDB code 4Y0W),^21^ we have recently explored the assembling mode of the YeaZ protein through computational studies, and we identified the protein‐mimetic tetradecapeptide sequence (**PMP1,** which corresponds to the sequence Arg73‐Leu86 of the *Pa*YeaZ protein) primarily responsible for the dimer formation within a shorter fragment of the α2 helical domain.^24^ In this work we explored the possibility of specifically inhibiting PAE growth modulating the formation of the macromolecular complex YgjD/YeaZ/YjeE by targeting the PPIs involved. The performed CAS analysis suggested that the main hot spots contributing to the YeaZ‐YeaZ dimer formation, which are also conserved in the YeaZ/YgjD heterodimer, are also important for the YeaZ‐**PMP1** interactions. In addition, MD calculations showed that **PMP1** conserved its binding to YeaZ during the whole simulation, maintaining the helical structure of the original α2 helix of the parent protein. Being **PMP1** too hydrophobic, we initially investigated the inhibitory effect of the previously synthesized helical peptide **PMP2**, which contains a slightly modified YeaZ binding sequence (Arg73 was replaced with an Ala) linked to the tetrapeptide KKKG tetrapeptide through a Gly‐Gly spacer. *In vitro* tests on the purified YeaZ protein provide evidence for the interaction between **PMP2** and YeaZ. The affinity of this interaction, unfortunately, could not be evaluated due to the poor solubility of **PMP2**. However, the growth of PAE cells and *E. coli* was not negatively affected by **PMP2** also at high concentrations, and we hypothesized that the peptide was not able to pass through the bacterial cell envelope. We therefore synthesized the peptide **PMP3**, where the tetradecapeptide Ala73‐Leu86 was conjugated to a CPP composed of four repeating units of Arg‐Ahx‐Arg, which was previously validated to be efficient in mediating the entry of PNAs within PAE. The *in vitro* tests on **PMP3** ability to bind YeaZ pointed out a binding with a low micromolar affinity constant. The tests on PAE cells showed a significant concentration‐dependent decrease in the growth rates not attributable to the CPP alone, suggesting that the conjugation with a suitable carrier can help to deliver hydrophobic sequences inside Gram‐negative cell envelope. Remarkably, the effects on *E. coli* cell growth were much weaker than those observed for PAE, which contrasts with the reported higher activity of free CPP on *E. coli* versus *P. aeruginosa*. This further highlights the importance of the specificity of the primary functional sequence (**PMP1**) designed to inhibit the targeted PPIs. In conclusion, our work represents the first example in the literature concerning the design and synthesis of peptide‐based inhibitors targeting PPIs of essential bacterial tRNA modification complex in PAE. We believe that the proof of concept here reported contributes to reinforcing the idea that targeting bacterial PPIs through suitable peptide sequences might represent a novel strategy to fight the increasing antibiotic resistance registered worldwide, and this work lays the foundation for future improvement.

## DISCLOSURE STATEMENT

The authors report no conflict of interest.

## Supporting information


**Scheme S1.** General SPPS procedure.

## References

[1] Stumpf MP , Thorne T , De Silva E , et al. Estimating the size of the human interactome. PNAS. 2008;105(19):6959‐6964. doi:10.1073/pnas.0708078105 18474861 PMC2383957

[2] Koh GC , Porras P , Aranda B , Hermjakob H , Orchard SE . Analyzing protein–protein interaction networks. J Proteome Res. 2012;11(4):2014‐2031. doi:10.1021/pr201211w 22385417

[3] Ivanov AA , Khuri FR , Fu H . Targeting protein–protein interactions as an anticancer strategy. Trends Pharmacol Sci. 2013;34(7):393‐400. doi:10.1016/j.tips.2013.04.007 23725674 PMC3773978

[4] Milroy LG , Grossmann TN , Hennig S , Brunsveld L , Ottmann C . Modulators of protein–protein interactions. Chem Rev. 2014;114(9):4695‐4748. doi:10.1021/cr400698c 24735440

[5] Lu H , Zhou Q , He J , et al. Recent advances in the development of protein–protein interactions modulators: mechanisms and clinical trials. Signal Transduct Target Ther. 2020;5(1):213. doi:10.1038/s41392-020-00315-3 32968059 PMC7511340

[6] Cabri W , Cantelmi P , Corbisiero D , et al. Therapeutic peptides targeting PPI in clinical development: overview, mechanism of action and perspectives. Front Mol Biosci. 2021;8:697586. doi:10.3389/fmolb.2021.697586 34195230 PMC8236712

[7] Coyne AG , Scott DE , Abell C . Drugging challenging targets using fragment‐based approaches. Curr Opin Chem Biol. 2010;14(3):299‐307. doi:10.1016/j.cbpa.2010.02.010 20223699

[8] Winter A , Higueruelo AP , Marsh M , Sigurdardottir A , Pitt WR , Blundell TL . Biophysical and computational fragment‐based approaches to targeting protein–protein interactions: applications in structure‐guided drug discovery. Q Rev Biophys. 2012;45(4):383‐426. doi:10.1017/S0033583512000108 22971516

[9] Contini A , Ferri N , Bucci R , et al. Peptide modulators of Rac1/Tiam1 protein‐protein interaction: an alternative approach for cardiovascular diseases. Peptide Sci. 2018;110(5):e23089. doi:10.1002/bip.23089 29178143

[10] Macut H , Hu X , Tarantino D , et al. Tuning PFKFB3 bisphosphatase activity through allosteric interference. Sci Rep. 2019;9(1):20333. doi:10.1038/s41598-019-56708-0 31889092 PMC6937325

[11] Cunningham AD , Qvit N , Mochly‐Rosen D . Peptides and peptidomimetics as regulators of protein–protein interactions. Curr Opin Struct Biol. 2017;44:59‐66. doi:10.1016/j.sbi.2016.12.009 28063303 PMC5496809

[12] Nevola L , Giralt E . Modulating protein–protein interactions: the potential of peptides. Chem Commun. 2015;51(16):3302‐3315. doi:10.1039/C4CC08565E 25578807

[13] Brown ED , Wright GD . Antibacterial drug discovery in the resistance era. Nature. 2016;529(7586):336‐343. doi:10.1038/nature17042 26791724

[14] Aggarwal R , Mahajan P , Pandiya S , et al. Antibiotic resistance: a global crisis, problems and solutions. Crit Rev Microbiol. 2024;17(5):1‐26. doi:10.1080/1040841X.2024.2313024 38381581

[15] Carro L . Protein–protein interactions in bacteria: a promising and challenging avenue towards the discovery of new antibiotics. Beilstein J Org Chem. 2018;14(1):2881‐2896. doi:10.3762/bjoc.14.267 30546472 PMC6278769

[16] Robinson A , J. Causer R , E. Dixon N . Architecture and conservation of the bacterial DNA replication machinery, an underexploited drug target. Curr Drug Targets. 2012;13(3):352‐372. doi:10.2174/138945012799424598 22206257 PMC3290774

[17] Global priority list of antibiotic‐resistant bacteria to guide research, discovery, and development of new antibiotics. Accessed February 2017. http://www.who.int/medicines/publications/WHO‐PPL‐Short_Summary_25Feb‐ET_NM_WHO.pdf?ua=1

[18] Serra A , Polese G , Braggion C , Rossi A . Non‐invasive proportional assist and pressure support ventilation in patients with cystic fibrosis and chronic respiratory failure. Thorax. 2002;57(1):50‐54. doi:10.1136/thorax.57.1.50 11809990 PMC1746175

[19] Crull MR , Somayaji R , Ramos KJ , et al. Changing rates of chronic Pseudomonas aeruginosa infections in cystic fibrosis: a population‐based cohort study. Clin Infect Dis. 2018;67(7):1089‐1095. doi:10.1093/cid/ciy215 29534149 PMC6137120

[20] Salsgiver EL , Fink AK , Knapp EA , et al. Changing epidemiology of the respiratory bacteriology of patients with cystic fibrosis. Chest. 2016;149(2):390‐400. doi:10.1378/chest.15-0676 26203598 PMC5831653

[21] Vecchietti D , Ferrara S , Rusmini R , Macchi R , Milani M , Bertoni G . Crystal structure of YeaZ from *Pseudomonas aeruginosa* . Biochem Biophys Res Commun. 2016;470(2):460‐465. doi:10.1016/j.bbrc.2016.01.008 26768361

[22] Thiaville PC , El Yacoubi B , Köhrer C , et al. Essentiality of threonylcarbamoyladenosine (t6 a), a universal *t*RNA modification, in bacteria. Mol Microbiol. 2015;98(6):1199‐1221. doi:10.1111/mmi.13209 26337258 PMC4963248

[23] Jacobs MA , Alwood A , Thaipisuttikul I , et al. Comprehensive transposon mutant library of *Pseudomonas aeruginosa* . PNAS. 2003;100(24):14339‐14344. doi:10.1073/pnas.2036282100 14617778 PMC283593

[24] Fasola E , Alboreggia G , Pieraccini S , et al. Conformational switch and multiple supramolecular structures of a newly identified self‐assembling protein‐mimetic peptide from *Pseudomonas aeruginosa* YeaZ protein. Front Chem. 2022;10:1038796. doi:10.3389/fchem.2022.1038796 36583150 PMC9792601

[25] Nichols CE , Lamb HK , Thompson P , et al. Crystal structure of the dimer of two essential *Salmonella typhimurium* proteins, YgjD & YeaZ and calorimetric evidence for the formation of a ternary YgjD–YeaZ–YjeE complex. Protein Sci. 2013;22(5):628‐640. doi:10.1002/pro.2247 23471679 PMC3649264

[26] Nichols CE , Johnson C , Lockyer M , et al. Structural characterization of *Salmonella typhimurium* YeaZ, an M22 O‐sialoglycoprotein endopeptidase homolog. Proteins: Struct Funct Bioinf. 2006;64(1):111‐123. doi:10.1002/prot.20982 16617437

[27] Ghosal A , Nielsen PE . Potent antibacterial antisense peptide–peptide nucleic acid conjugates against *Pseudomonas aeruginosa* . Nucleic Acid Ther. 2012;22(5):323‐334. doi:10.1089/nat.2012.0370 23030590 PMC3464458

[28] Lindorff‐Larsen K , Piana S , Palmo K , et al. Improved side‐chain torsion potentials for the Amber ff99SB protein force field. Proteins: Struct Funct Bioinf. 2010;78(8):1950‐1958. doi:10.1002/prot.22711 PMC2970904 20408171

[29] Jorgensen WL , Chandrasekhar J , Madura JD , Impey RW , Klein ML . Comparison of simple potential functions for simulating liquid water. J Chem Phys. 1983;79(2):926‐935. doi:10.1063/1.445869

[30] Bussi G , Donadio D , Parrinello M . Canonical sampling through velocity rescaling. J Chem Phys. 2007;126(1):014101. doi:10.1063/1.2408420 17212484

[31] Berendsen HJ , Postma JV , Van Gunsteren WF , Di Nola AR , Haak JR . Molecular dynamics with coupling to an external bath. J Chem Phys. 1984;81(8):3684‐3690. doi:10.1063/1.448118

[32] Darden T , York D , Pedersen L . Particle mesh Ewald: An N·log(N) method for Ewald sums in large systems. J Chem Phys. 1993;98(12):10089‐10092. doi:10.1063/1.464397

[33] Hess B , Bekker H , Berendsen HJ , Fraaije JG . LINCS: a linear constraint solver for molecular simulations. J Comput Chem. 1997;18(12):1463‐1472. doi:10.1002/(SICI)1096-987X(199709)18:12<1463::AID-JCC4>3.0.CO;2-H

[34] Van Der Spoel D , Lindahl E , Hess B , Groenhof G , Mark AE , Berendsen HJ . GROMACS: fast, flexible, and free. J Comput Chem. 2005;26(16):1701‐1718. doi:10.1002/jcc.20291 16211538

[35] Massova I , Kollman PA . Combined molecular mechanical and continuum solvent approach (MM‐PBSA/GBSA) to predict ligand binding. Perspect Drug Discov Des. 2000;18(1):113‐135. doi:10.1023/A:1008763014207

[36] Paissoni C , Spiliotopoulos D , Musco G , Spitaleri A . GMXPBSA 2.0: a GROMACS tool to perform MM/PBSA and computational alanine scanning. Comput Phys Commun. 2014;185(11):2920‐2929. doi:10.1016/j.cpc.2014.06.019

[37] Stover CK , Pham XQ , Erwin AL , et al. Complete genome sequence of *Pseudomonas aeruginosa* PAO1, an opportunistic pathogen. Nature. 2000;406(6799):959‐964. doi:10.1038/35023079 10984043

[38] Blattner FR , Plunkett G III , Bloch CA , et al. The complete genome sequence of *Escherichia coli* K‐12. Science. 1997;277(5331):1453‐1462. doi:10.1126/science.277.5331.1453 9278503

[39] Barreto‐Santamaría A , Arévalo‐Pinzón G , Patarroyo MA , Patarroyo ME . How to combat gram‐negative bacteria using antimicrobial peptides: a challenge or an unattainable goal? Antibiotics. 2021;10(12):1499. doi:10.3390/antibiotics10121499 34943713 PMC8698890

[40] Komin A , Russell LM , Hristova KA , Searson PC . Peptide‐based strategies for enhanced cell uptake, transcellular transport, and circulation: mechanisms and challenges. Adv Drug Deliv Rev. 2017;110:52‐64. doi:10.1016/j.addr.2016.06.002 27313077

[41] Bechara C , Sagan S . Cell‐penetrating peptides: 20 years later, where do we stand? FEBS Lett. 2013;587(12):1693‐1702. doi:10.1016/j.febslet.2013.04.031 23669356

[42] Rothbard JB , Kreider E , VanDeusen CL , Wright L , Wylie BL , Wender PA . Arginine‐rich molecular transporters for drug delivery: role of backbone spacing in cellular uptake. J Med Chem. 2002;45(17):3612‐3618. doi:10.1021/jm0105676 12166934

[43] Amirkhanov NV , Tikunova NV , Pyshnyi DV . Synthetic antimicrobial peptides: I. Antimicrobial activity of amphiphilic and nonamphiphilic cationic peptides. Russ J Bioorg Chem. 2018;44(5):492‐503. doi:10.1134/S1068162018050035

[44] Vincenzi M , Mercurio FA , Leone M . About TFE: old and new findings. Curr Protein Pept Sci. 2019;20(5):425‐451. doi:10.2174/1389203720666190214152439 30767740

[45] Teplova M , Tereshko V , Sanishvili R , et al. The structure of the yrdC gene product from *Escherichia coli* reveals a new fold and suggests a role in RNA binding. Protein Sci. 2000;9(12):2557‐2566. doi:10.1110/ps.9.12.2557 11206077 PMC2144518

