# Supplementary material for: Impairing protein–protein interactions in an essential tRNA modification complex: An innovative antimicrobial strategy against Pseudomonas aeruginosa
Source: J Pept Sci. 2024 Oct 22;31(2):e3658. doi: 10.1002/psc.3658 (PMC11669939; doi:10.1002/psc.3658)

**SUPPLEMENTARY MATERIAL**

**Impairing protein-protein interactions in an essential tRNA modification complex: an innovative antimicrobial strategy against *Pseudomonas aeruginosa***

Michela Bollati^†a,d^, Elettra Fasola^†b^, Stefano Pieraccini^c^, Francesca Freddi^d^, Paolo Cocomazzi^a^, Francesco Oliva^c^, Merlin Klußmann,^e^ Angelo Maspero^b^, Umberto Piarulli^b^, Silvia Ferrara^a,d^, Sara Pellegrino^f^, Giovanni Bertoni^d^*, Silvia Gazzola^b^*

*^a^Institute of Biophysics, National Research Council, Milan, Italy*

*^b^Department of Science and High Technology, Università degli Studi dell’Insubria, Como, Italy*

*^c^Department of Chemistry, Università degli Studi di Milano, Milan, Italy*

*^d^Department of Biosciences, Università degli Studi di Milano, Milan, Italy*

*^e^Department of Chemistry, Institute for Biochemistry, University of Cologne, D-50674 Cologne, Germany*

*^f^Pharmaceutical Science Department, University of Milan, IT-20133 Milan, Italy*

^†^ The authors MB and EF have contributed equally to the work.

**Correspondence:**

Silvia Gazzola, Department of Science and High Technology, Università degli Studi dell’Insubria, Via Valleggio, 9, 22100, Como (Italy). E-mail: [s.gazzola@uninsubria.it](mailto:s.gazzola@uninsubria.it)

Giovanni Bertoni, Department of Biosciences, Università degli Studi di Milano, Via Celoria, 26, 20133 Milan (Italy). E-mail: [giovanni.bertoni@unimi.it](mailto:giovanni.bertoni@unimi.it)

**PEPTIDE SYNTHESIS AND CHARACTERIZATION**

**Solid phase peptide synthesis procedures**

Peptides were synthesized by Solid Phase Peptide Synthesis as reported in **Scheme 1**, affording yields of 60% for **PMP2** and 7% for **PMP3** due to triple preparative HPLC purification, and the final purity of peptides was ≥95%.

**Supplementary Scheme 1** – General SPPS procedure.

Peptides were analyzed by analytical RP-HPLC SHIMADZU LC-20AP equipped with diode array UV detector and C-18 column.

**Flux**: 1 mL/min

**UV wavelength:** 199-201 nm

**Solvent A:** H_2_O milliQ, 0.1% HCOOH

**Solvent B:** CH_3_CN

**Gradient:** isocratic 98% A–2% B for 5 minutes; from 98% A–2% B to 75% A–25% B in 5 minutes; from 75% A–25% B to 30% A–70% B in 5 minutes; isocratic 30% A–70% B for 7 minutes.

- **Compound PMP2**

Chemical Formula: C_149_H_269_N_55_O_32_

Exact mass = 1897.1513

**PMP2** synthesis and characterization were previously reported (Fasola E, Alboreggia G, Pieraccini S, et al. *Front. Chem.* **2022**; *10*: 1038796).

- **Compound PMP3**


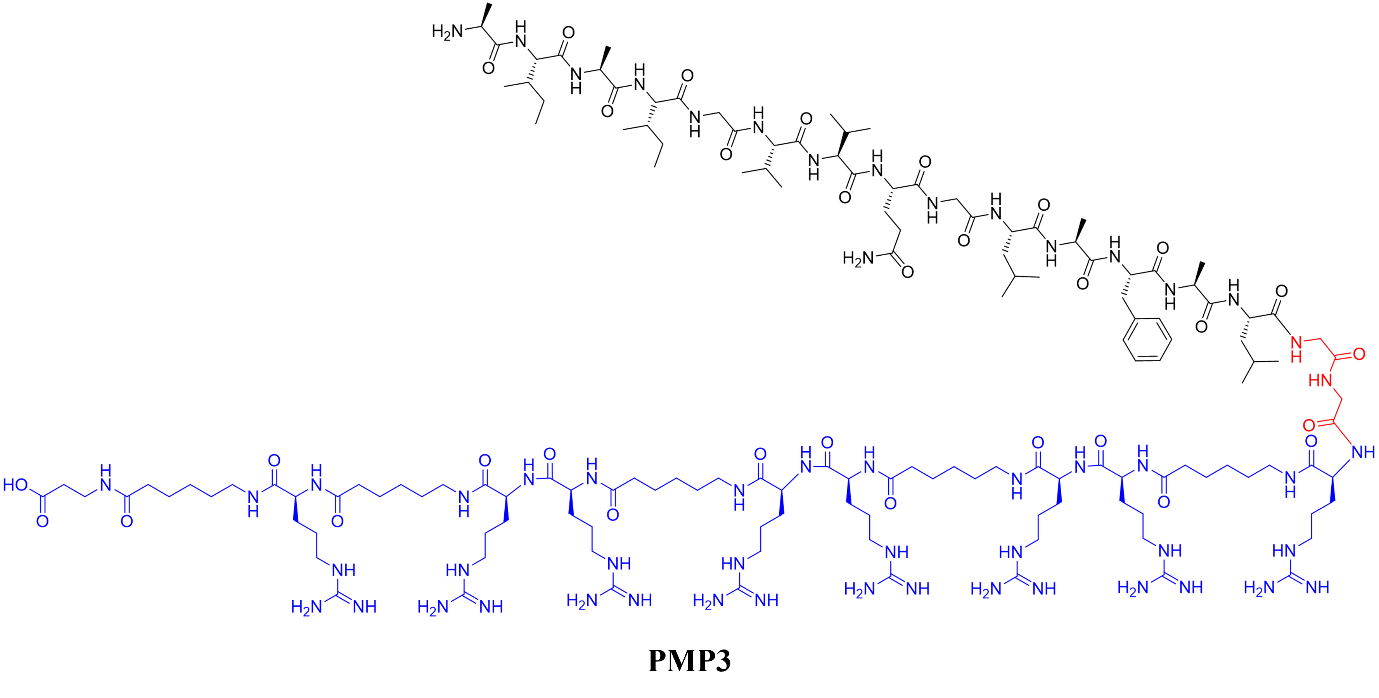


Chemical Formula: C_149_H_269_N_55_O_32_

Exact mass = 3341.1113

***HPLC trace of PMP3***

**PMP3** was analyzed with the following gradient and conditions:

Flux: 1 mL/min

UV wavelength: 254 nm

Solvent A: H_2_O milliQ + 0.1% TFA; Solvent B: CH_3_CN

Gradient: isocratic 2% B for 5 minutes; from 2%B to 25%B in 5 minutes; from 25%B to 70%B in 5 minutes; isocratic 30%A–70%B for 5 minutes.

Blue traces: **PMP3** elution

Grey traces: Blank elution

***HRMS spectra of PMP3***


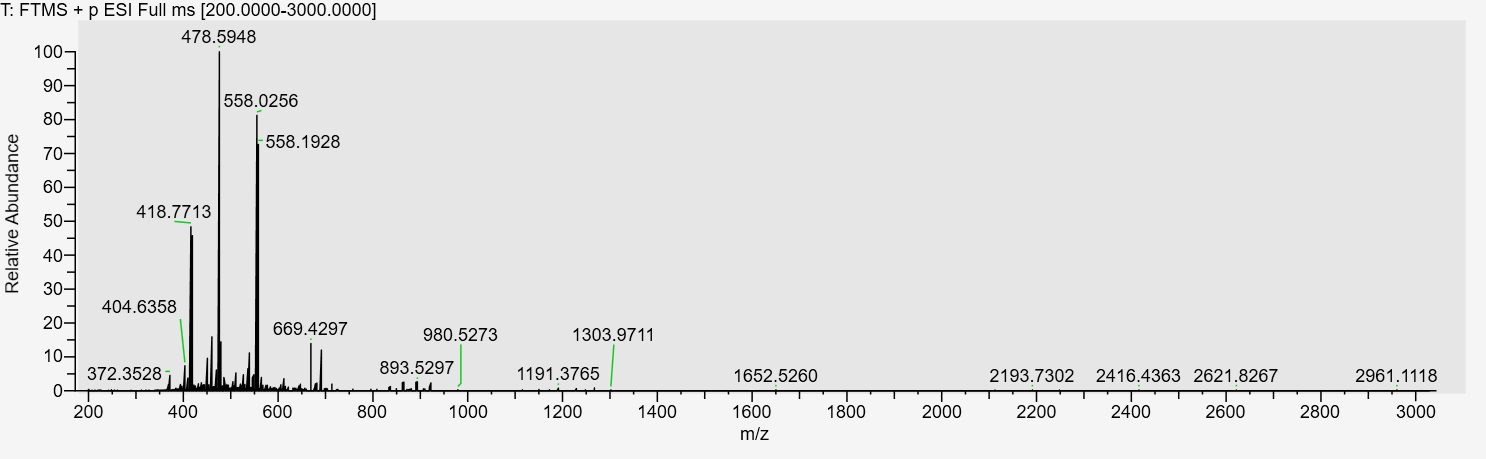


m/z [M+5H]^5+^: found = 669.4297; m/z [M+6H]^6+^: found = 558.0256; m/z [M+7H]^7+^: found = 478.5948; m/z [M+8H]^8+^: found = 418.7713; m/z [M+9H]^9+^: found = 372.3528

- **CPP**

Chemical Formula: C_81_H_158_N_38_O_15_

Exact mass = 1903.88

***HPLC trace of CPP***

**CPP** was analyzed with the following gradient and conditions:

Flux: 1 mL/min

UV wavelength: 254 nm

Solvent A: H_2_O milliQ + 0.1% TFA; Solvent B: CH_3_CN + 0.1% TFA

Gradient: 5% B for 2 minutes, from 5% to 80% B in 25 min.


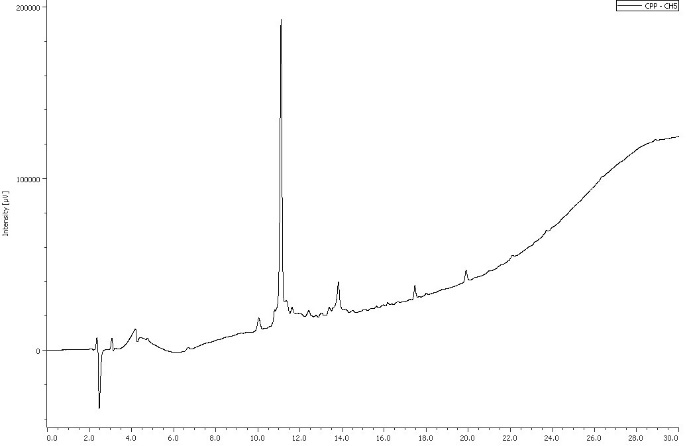


***HRMS spectra of CPP***


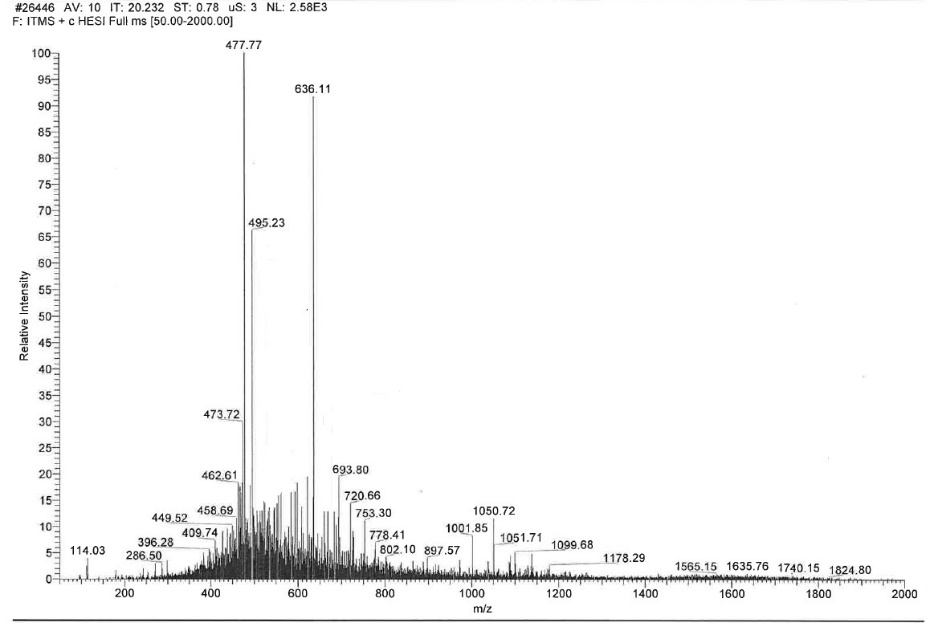


m/z [M+3H]^3+^: found = 636.11; m/z [M+4H]^4+^: found = 477.77; m/z [M+4H+Na^+^]^4+^: found = 495.23

***CIRCULAR DICHROISM SPECTRA OF CPP***

***K_d_ DETERMINATION BY MICROSCALE THERMOFORESIS***


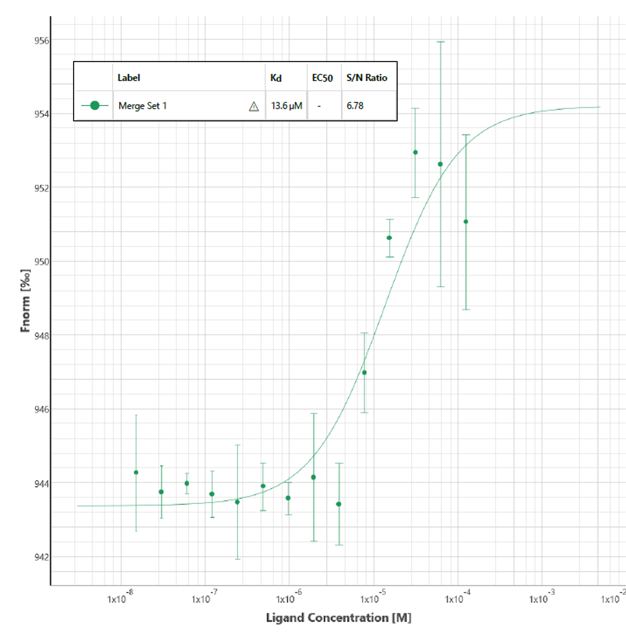

Supplement: Supplementary file 1 — Scheme S1. General SPPS procedure. [file PSC-31-e3658-s001.docx]
